# Artificial Intelligence-Assisted Endoscopic Ultrasound-Guided Ablation of Pancreatic Neuroendocrine Tumors: Toward Precision Diagnosis, Risk Stratification, and Personalized Therapy

**DOI:** 10.7759/cureus.109721

**Published:** 2026-05-27

**Authors:** Ahmed Salman, Ahmed Elewa, Ahmed Safina, Ahmed Marwan

**Affiliations:** 1 Department of Internal Medicine, Faculty of Medicine, Cairo University, Cairo, EGY; 2 Department of General Surgery, National Hepatology and Tropical Medicine Research Institute, Cairo, EGY; 3 Department of General Surgery, Kasralainy School of Medicine, Cairo University, Cairo, EGY; 4 Department of Internal Medicine, Faculty of Medicine, Mansoura University, Mansoura, EGY

**Keywords:** artificial intelligence, endoscopic ultrasound, pancreatic neuroendocrine tumor, precision endoscopy, radiofrequency ablation

## Abstract

Pancreatic neuroendocrine tumors (pNETs) are increasingly detected at an early stage because of the wider use of cross-sectional imaging and endoscopic ultrasound. Their management remains challenging, particularly for small functioning tumors and selected non-functioning lesions, where the risks of pancreatic surgery must be balanced against tumor biology, symptoms, progression risk, and patient preference. Endoscopic ultrasound (EUS)-guided ablation, particularly radiofrequency ablation, has emerged as a minimally invasive, organ-preserving option for carefully selected patients with small pNETs, especially insulinomas and low-risk non-functioning lesions. However, current evidence is limited by small cohorts, heterogeneous techniques, variable follow-up protocols, and uncertainty regarding long-term oncological outcomes. Artificial intelligence (AI) may enhance this evolving field by supporting EUS-based lesion detection, characterization, grading prediction, risk stratification, patient selection, procedural planning, and post-ablation surveillance. AI-assisted models using EUS images, radiomics, pathology, and multimodal clinical data may help identify patients most likely to benefit from ablation while avoiding inappropriate local therapy in biologically aggressive disease. This review summarizes the current role of EUS-guided ablation for pNETs and explores the emerging potential of AI to support precision diagnosis, individualized risk assessment, and personalized minimally invasive therapy.

## Introduction and background

Pancreatic neuroendocrine tumors (pNETs) are uncommon but increasingly recognized neoplasms arising from the endocrine cells of the pancreas. Although they account for only a small proportion of pancreatic tumors, their clinical relevance has expanded because of rising detection rates, the widespread use of high-resolution cross-sectional imaging, and increased recognition of small incidentally discovered lesions [1]. This epidemiological shift has created a growing management dilemma. While some pNETs behave indolently and may be suitable for surveillance, others demonstrate clinically significant malignant potential, hormonal activity, local progression, or metastatic dissemination. Consequently, contemporary management requires careful integration of tumor size, functional status, grade, growth kinetics, anatomical location, patient comorbidity, and treatment preference [2,3].

Surgical resection remains the standard curative treatment for many localized pNETs, particularly functioning tumors and lesions with features suggesting aggressive biology. However, pancreatic surgery is associated with relevant morbidity, including pancreatic fistula, delayed gastric emptying, endocrine or exocrine insufficiency, and procedure-specific risks depending on lesion location. These concerns are especially important for small pNETs, where the biological risk of the tumor may be modest while the morbidity of surgery may be disproportionate. For selected small non-functioning pNETs, guidelines and expert reviews increasingly recognize that active surveillance may be appropriate, although this approach can be limited by uncertainty regarding long-term progression and patient anxiety [2,3].

Endoscopic ultrasound (EUS) occupies a central role in the evaluation of pNETs because it provides high-resolution imaging of the pancreas and enables precise assessment of lesion size, morphology, vascular relationship, and proximity to the pancreatic duct. EUS-guided tissue acquisition further allows histological confirmation and Ki-67 assessment, which are essential for grading and risk stratification [4,5]. Nevertheless, EUS-guided sampling has limitations, including sampling error, tumor heterogeneity, and potential underestimation of grade when compared with surgical specimens. These limitations highlight the need for improved tools that can refine pre-treatment risk assessment and guide individualized decision-making [5].

In this context, EUS-guided ablation has emerged as a minimally invasive therapeutic option for carefully selected patients with pNETs. Radiofrequency ablation, performed under real-time EUS guidance, allows targeted thermal destruction of pancreatic lesions while avoiding major surgery. Early studies and systematic reviews suggest promising technical and clinical outcomes, particularly for functioning insulinomas and selected small non-functioning pNETs in patients who are poor surgical candidates or who decline surgery [6,7]. However, the current evidence remains limited by small cohorts, heterogeneous techniques, variable definitions of clinical success, and limited long-term oncological follow-up.

Artificial intelligence (AI) may provide the next step in the evolution of EUS-based pNET management. AI-assisted analysis of EUS images, radiomics, and multimodal clinical data may help improve lesion characterization, predict histopathological grade, identify higher-risk tumors, and support selection between surveillance, surgery, and EUS-guided ablation [8]. Rather than replacing expert endosonographers, AI may function as a decision-support tool that enhances consistency, objectivity, and precision across the diagnostic and therapeutic pathway.

This focused narrative review is intended to synthesize current evidence and emerging concepts and discusses the emerging concept of AI-assisted EUS-guided ablation of pNETs, focusing on its potential role in precision diagnosis, risk stratification, patient selection, procedural planning, and post-ablation surveillance. The aim is to frame EUS-guided ablation not merely as a technical intervention but as part of a broader precision-endoscopy strategy for personalized management of pNETs.

This narrative review was developed through a focused literature search of PubMed/Medical Literature Analysis and Retrieval System Online (MEDLINE), Scopus, and Google Scholar. The search included combinations of the following terms: “pancreatic neuroendocrine tumor,” “pNET,” “endoscopic ultrasound,” “EUS-guided ablation,” “radiofrequency ablation,” “insulinoma,” “artificial intelligence,” “deep learning,” “radiomics,” “Ki-67,” “tumor grading,” and “risk stratification.” The search focused on English-language articles relevant to EUS diagnosis, EUS-guided ablation, AI-assisted EUS, radiomics-based grading, and personalized management of pNETs.

Priority was given to recent systematic reviews, meta-analyses, prospective or multicenter studies, guideline-oriented publications, and original studies directly relevant to EUS-guided therapy or AI-based pancreatic imaging. Foundational studies were also included when they provided important background on EUS diagnosis, Ki-67 grading, or pNET management. This review was intended as a focused narrative synthesis rather than a systematic review; therefore, formal PRISMA methodology, risk-of-bias assessment, and quantitative evidence synthesis were not performed.

## Review

pNETs are uncommon but increasingly recognized neoplasms arising from the endocrine cells of the pancreas. Although they account for only a small proportion of pancreatic tumors, their clinical relevance has expanded because of rising detection rates, the widespread use of high-resolution cross-sectional imaging, and increased recognition of small incidentally discovered lesions [1]. This epidemiological shift has created a growing management dilemma. While some pNETs behave indolently and may be suitable for surveillance, others demonstrate clinically significant malignant potential, hormonal activity, local progression, or metastatic dissemination. Consequently, contemporary management requires careful integration of tumor size, functional status, grade, growth kinetics, anatomical location, patient comorbidity, and treatment preference [2,3].

Surgical resection remains the standard curative treatment for many localized pNETs, particularly functioning tumors and lesions with features suggesting aggressive biology. However, pancreatic surgery is associated with relevant morbidity, including pancreatic fistula, delayed gastric emptying, endocrine or exocrine insufficiency, and procedure-specific risks depending on lesion location. These concerns are especially important for small pNETs, where the biological risk of the tumor may be modest while the morbidity of surgery may be disproportionate. For selected small non-functioning pNETs, guidelines and expert reviews increasingly recognize that active surveillance may be appropriate, although this approach can be limited by uncertainty regarding long-term progression and patient anxiety [2,3].

EUS occupies a central role in the evaluation of pNETs because it provides high-resolution imaging of the pancreas and enables precise assessment of lesion size, morphology, vascular relationship, and proximity to the pancreatic duct. EUS-guided tissue acquisition further allows histological confirmation and Ki-67 assessment, which are essential for grading and risk stratification [4,5]. Nevertheless, EUS-guided sampling has limitations, including sampling error, tumor heterogeneity, and potential underestimation of grade when compared with surgical specimens. These limitations highlight the need for improved tools that can refine pre-treatment risk assessment and guide individualized decision-making [5].

In this context, EUS-guided ablation has emerged as a minimally invasive therapeutic option for carefully selected patients with pNETs. Radiofrequency ablation, performed under real-time EUS guidance, allows targeted thermal destruction of pancreatic lesions while avoiding major surgery. Early studies and systematic reviews suggest promising technical and clinical outcomes, particularly for functioning insulinomas and selected small non-functioning pNETs in patients who are poor surgical candidates or who decline surgery [6,7]. However, the current evidence remains limited by small cohorts, heterogeneous techniques, variable definitions of clinical success, and limited long-term oncological follow-up.

AI may provide the next step in the evolution of EUS-based pNET management. AI-assisted analysis of EUS images, radiomics, and multimodal clinical data may help improve lesion characterization, predict histopathological grade, identify higher-risk tumors, and support selection between surveillance, surgery, and EUS-guided ablation [8]. Rather than replacing expert endosonographers, AI may function as a decision-support tool that enhances consistency, objectivity, and precision across the diagnostic and therapeutic pathway.

This focused narrative review is intended to synthesize current evidence and emerging concepts and discusses the emerging concept of AI-assisted EUS-guided ablation of pNETs, focusing on its potential role in precision diagnosis, risk stratification, patient selection, procedural planning, and post-ablation surveillance. The aim is to frame EUS-guided ablation not merely as a technical intervention, but as part of a broader precision-endoscopy strategy for personalized management of pNETs.

This narrative review was developed through a focused literature search of PubMed/MEDLINE, Scopus, and Google Scholar. The search included combinations of the following terms: “pancreatic neuroendocrine tumor,” “pNET,” “endoscopic ultrasound,” “EUS-guided ablation,” “radiofrequency ablation,” “insulinoma,” “artificial intelligence,” “deep learning,” “radiomics,” “Ki-67,” “tumor grading,” and “risk stratification.” The search focused on English-language articles relevant to EUS diagnosis, EUS-guided ablation, AI-assisted EUS, radiomics-based grading, and personalized management of pNETs.

Priority was given to recent systematic reviews, meta-analyses, prospective or multicenter studies, guideline-oriented publications, and original studies directly relevant to EUS-guided therapy or AI-based pancreatic imaging. Foundational studies were also included when they provided important background on EUS diagnosis, Ki-67 grading, or pNETs management. This review was intended as a focused narrative synthesis rather than a systematic review; therefore, formal PRISMA methodology, risk-of-bias assessment, and quantitative evidence synthesis were not performed.

Current role of EUS in pNETs

EUS has emerged as an essential modality in the diagnostic and therapeutic assessment of pNETs because of its ability to provide high-resolution imaging of the pancreas from the stomach and duodenum. This is particularly important for small pNETs, including insulinomas, which may be difficult to detect on cross-sectional imaging. In a systematic review and meta-analysis, EUS demonstrated high diagnostic accuracy for pNETs, with particularly strong performance for insulinoma localization. Earlier clinical series similarly supported EUS as a primary diagnostic modality in patients with suspected pNETs, reporting high sensitivity and accuracy for lesion detection. These features make EUS especially valuable when conventional imaging is negative, equivocal, or insufficient for treatment planning [9,10].

Beyond lesion detection, EUS provides detailed anatomical information that is directly relevant to management. It allows assessment of tumor size, echogenicity, margins, vascularity, and relationship to the pancreatic duct, bile duct, and adjacent vascular structures. These features are important not only for diagnosis but also for determining whether a lesion may be suitable for local therapy. For EUS-guided ablation, technical feasibility depends on accurate lesion localization, safe needle access, avoidance of intervening vessels, and sufficient distance from the main pancreatic duct when possible. Therefore, EUS serves as both a diagnostic and pre-therapeutic staging tool [9,11].

EUS-guided tissue acquisition further strengthens the role of EUS in pNET management. Fine-needle aspiration and fine-needle biopsy can provide cytopathological or histopathological confirmation and allow assessment of immunohistochemical markers, including chromogranin A, synaptophysin, and Ki-67. Ki-67 is particularly important because tumor grade is a major determinant of prognosis and treatment strategy. Several studies have evaluated the concordance between Ki-67 assessment of EUS-guided samples and surgical specimens, showing that EUS-guided tissue acquisition can provide clinically useful grading information, although discordance remains possible due to sampling error and intratumoral heterogeneity [11-13].

These limitations are clinically relevant in the context of personalized therapy. For small non-functioning pNETs, decisions among surveillance, surgery, and local ablation often depend on an accurate estimate of biological risk. An underestimated Ki-67 index may lead to inappropriate surveillance or local therapy, whereas overestimation may expose a patient to unnecessary surgery. Similarly, lesion size alone may be insufficient to determine risk, particularly for tumors in the 1-2 cm range, where management remains individualized. As a result, EUS findings must be interpreted in conjunction with cross-sectional imaging, pathology, tumor function, growth kinetics, patient comorbidities, and multidisciplinary assessment [2,3,13].

The evolving role of EUS is therefore broader than diagnosis alone. In the modern management of pNETs, EUS functions as a platform for localization, tissue acquisition, risk assessment, therapeutic planning, and image-guided intervention. This integrated role provides the foundation for EUS-guided ablation and creates a logical opportunity for AI to enhance diagnostic consistency, improve grading prediction, and support patient selection for minimally invasive therapy [6,8,13].

EUS-guided ablation of pNETs

EUS-guided ablation has emerged as a minimally invasive therapeutic option for selected patients with pNETs, particularly those with small functioning tumors or small non-functioning lesions in whom surgery may be undesirable, high risk, or disproportionate to tumor biology. The two main ablative approaches described for pNETs are ethanol injection and radiofrequency ablation, although EUS-guided radiofrequency ablation has gained increasing attention because it allows controlled thermal destruction under real-time EUS guidance. Current evidence suggests that EUS-guided ablation is technically feasible and clinically promising, but it remains best considered within expert centers and multidisciplinary decision-making pathways because long-term oncological outcomes are still limited [14,15].

The technique of EUS-guided radiofrequency ablation involves careful lesion localization, Doppler assessment to avoid intervening vessels, and placement of a dedicated RFA needle or electrode into the target tumor under EUS guidance. Energy is then delivered to induce coagulative necrosis, often accompanied by echogenic changes within the lesion during ablation. Technical planning is essential because the safety of the procedure depends on tumor size, accessibility, distance from the main pancreatic duct, proximity to vessels, and the ability to achieve a stable needle position. These factors are particularly important in pancreatic lesions, where thermal injury may theoretically cause pancreatitis, ductal damage, bleeding, or peripancreatic inflammation [14,16].

The strongest clinical rationale for EUS-guided ablation currently exists for insulinomas. These tumors are often small and benign but can cause significant morbidity due to recurrent hypoglycemia. Surgical resection is effective, but may be associated with pancreatic morbidity, particularly when lesions are deeply located or require formal pancreatic resection. EUS-guided radiofrequency ablation offers a potential organ-preserving alternative in patients who are poor surgical candidates, refuse surgery, or have lesions suitable for safe local ablation. In published series, EUS-guided radiofrequency ablation has been associated with symptomatic improvement and biochemical control in many patients with insulinoma, although recurrence and need for repeat intervention remain important considerations [16,17].

For small non-functioning pNETs, the role of EUS-guided ablation is more nuanced. Many small non-functioning tumors demonstrate indolent behavior and may be managed with surveillance, while others may progress or reveal higher-risk biology. EUS-radiofrequency ablation may therefore be considered in selected patients with small, localized, low-grade lesions when surveillance is undesirable or surgery is high risk. However, compared with insulinomas, the therapeutic endpoint is less straightforward because symptom resolution cannot be used as a clinical marker of success. Instead, response assessment depends on imaging changes, reduction in lesion size or enhancement, absence of progression, and long-term recurrence monitoring [15,18].

Safety remains central to the clinical adoption of EUS-guided ablation. Reported adverse events include abdominal pain, mild pancreatitis, fever, bleeding, and peripancreatic fluid collection, although most published events are mild or moderate. Available comparative data suggest that EUS-guided ablation may reduce short-term morbidity and hospital stay compared with surgery in selected insulinoma patients, but may carry a higher risk of recurrence, emphasizing that ablation should not be viewed as a universal replacement for surgery. Instead, it should be positioned as a personalized option for carefully selected patients after consideration of tumor biology, technical feasibility, patient preference, and local expertise [17,18].

Overall, EUS-guided ablation represents an important step toward less invasive management of pNETs. Its greatest current value appears to be in functioning insulinomas and selected small non-functioning pNETs where surgery is either high risk or potentially excessive. Nevertheless, the evidence base is still evolving, and major limitations include small sample sizes, heterogeneous ablation protocols, variable follow-up duration, inconsistent response definitions, and limited prospective comparative data. These gaps create a clear opportunity for AI to improve lesion characterization, risk prediction, patient selection, procedural planning, and surveillance after ablation [14,15,18].

AI in EUS-based diagnosis of pancreatic lesions

AI is increasingly being investigated as a tool to enhance EUS-based diagnosis of pancreatic lesions. Conventional EUS is highly operator dependent, and diagnostic accuracy may vary according to lesion size, image quality, endosonographer experience, and availability of tissue confirmation. AI-based systems, particularly those using convolutional neural networks and deep learning, can analyze visual patterns within EUS images and may identify subtle features that are difficult to recognize consistently by human interpretation alone. In the context of pancreatic disease, most AI-EUS research has focused on solid pancreatic lesions, pancreatic cancer, and pancreatic cystic lesions, but these developments provide the technical foundation for more specific applications to pNETs [19,20]. However, much of the current AI-EUS evidence is derived from studies of solid pancreatic lesions in general, particularly pancreatic cancer, and should not be interpreted as direct evidence for pNET-specific ablation decision-making.

Several studies have shown that AI-assisted EUS may improve the detection and classification of focal pancreatic lesions. Real-time computer-aided diagnosis systems have been developed to distinguish pancreatic cancer from other focal pancreatic masses using EUS imaging, while more recent multimodal AI models have incorporated clinical and imaging information to improve diagnostic performance. These approaches are relevant to pNETs because small neuroendocrine tumors may mimic other solid pancreatic lesions, including pancreatic adenocarcinoma, solid pseudopapillary neoplasms, and focal inflammatory masses. Improved AI-assisted differentiation may therefore reduce diagnostic uncertainty and guide appropriate tissue acquisition and therapeutic planning [21,22].

For pNETs specifically, AI may help standardize EUS interpretation by supporting lesion recognition, segmentation, and characterization. pNETs often appear as well-demarcated, hypoechoic, hypervascular lesions, but their appearance can vary according to size, grade, fibrosis, cystic change, necrosis, or prior treatment. AI-based image analysis may allow automated extraction of quantitative imaging features, including texture, margins, echogenicity, vascular patterns, and spatial relationships. These features may eventually support differentiation between pNETs and other solid pancreatic lesions and provide more reproducible assessment across different operators and centers [23,24].

Deep learning and radiomics are particularly relevant because they move beyond subjective visual interpretation. Radiomics converts medical images into quantitative data, while deep learning models can automatically learn complex image features from large datasets. In EUS, these techniques may be applied to still images, video sequences, contrast-enhanced EUS, elastography, or multimodal datasets that combine EUS with CT, MRI, pathology, and clinical variables. This approach is attractive for pNETs because management depends not only on whether a lesion is present, but also on whether it is likely to behave indolently or aggressively [20,24].

Despite this promise, AI-assisted EUS diagnosis remains at an early stage. Many available studies are retrospective, single-center, and based on relatively small or selected image datasets. External validation is limited, and performance may decline when algorithms are tested across different EUS processors, imaging settings, operators, and institutions. In addition, most AI-EUS studies have evaluated diagnostic classification rather than clinically meaningful outcomes such as progression, recurrence, metastatic risk, or response to ablation. Therefore, AI should currently be viewed as an adjunct to expert EUS interpretation and multidisciplinary assessment rather than an independent decision-making tool [19,20,22].

AI for grading and risk stratification of pNETs

Accurate grading and risk stratification are central to the management of pNETs because biological behavior varies widely between indolent low-grade lesions and more aggressive tumors with metastatic potential. The Ki-67 proliferation index and mitotic count form the basis of current grading systems and strongly influence decisions regarding surveillance, surgery, systemic therapy, and suitability for local treatment. In the context of EUS-guided ablation, this distinction is particularly important because local therapy is most appropriate for carefully selected lesions with favorable biology, whereas higher-grade or clinically aggressive tumors usually require surgical or systemic oncological strategies [25,26].

Although EUS-guided tissue acquisition can provide pre-treatment grading information, Ki-67 assessment is imperfect because of tumor heterogeneity, small sample size, sampling error, and interobserver variability in pathological interpretation. Discordance between EUS-derived Ki-67 grading and surgical specimen grading may occur, particularly when the sampled area does not represent the most proliferative component of the tumor. These limitations are clinically relevant for therapeutic decision-making because underestimation of grade may result in inappropriate surveillance or local ablation, while overestimation may lead to unnecessary surgical treatment. Therefore, additional non-invasive or minimally invasive tools that improve grade prediction would be valuable [11-13,27].

AI-assisted analysis of EUS images offers a promising approach to pre-treatment grading of pNETs. A recent pilot study evaluated deep-learning analysis of EUS images for predicting the histopathological grade of pNETs and suggested that AI-based image interpretation may help distinguish tumor grade categories. Although early and requiring external validation, this work is important because it directly links AI, EUS, and pNET biology, rather than focusing only on lesion detection. If confirmed in larger multicenter datasets, such models could support endosonographers by identifying imaging patterns associated with higher-risk pathology [8,28].

Radiomics may further enhance risk stratification by converting imaging data into quantitative features that reflect tumor texture, heterogeneity, margins, vascularity, enhancement pattern, and spatial relationships. Studies using CT, MRI, and ultrasound-based radiomics have reported potential value in predicting pNET grade and Ki-67 category. Although most of this evidence is derived from cross-sectional imaging rather than EUS, it supports the broader principle that imaging phenotypes can correlate with tumor biology. Future models may combine EUS radiomics with CT or MRI radiomics to generate more robust multimodal predictions of grade and progression risk [29-31]. It is important to note that several radiomics studies in pNET grading are based on CT, MRI, or transabdominal ultrasound rather than EUS. Therefore, these findings support the broader biological plausibility of imaging-based risk prediction, but they cannot yet be directly translated into EUS-guided ablation selection without dedicated EUS-based validation.

The value of AI in pNET risk stratification may be greatest when multiple data streams are integrated. A clinically useful model would not rely on EUS appearance alone, but would combine tumor size, location, enhancement pattern, duct or vessel proximity, functional status, growth kinetics, EUS-guided histology, Ki-67 index, patient comorbidity, and cross-sectional imaging features. Such a multimodal approach could generate individualized risk estimates and support decisions between active surveillance, surgery, and EUS-guided ablation. In this way, AI may help shift management from a size-based approach toward a more biologically informed and personalized strategy [22,29,31].

However, several barriers must be addressed before AI-based grading or risk prediction can be adopted clinically. Most available models are retrospective, trained on limited datasets, and lack external validation across institutions, EUS platforms, and operators. There are also concerns regarding algorithmic bias, explainability, reproducibility, and integration into real-time EUS workflows. Importantly, predictive performance must be evaluated against clinically meaningful outcomes such as progression, recurrence, metastatic development, need for surgery, and post-ablation failure, rather than image classification alone. Until such evidence is available, AI should be considered a supportive tool that complements histology, imaging, and multidisciplinary clinical judgment [19,20,24].

AI-assisted patient selection, procedural planning, and surveillance

The most clinically relevant role of AI in this field may be its potential to improve patient selection for EUS-guided ablation. Current decisions are usually based on tumor size, functional status, grade, anatomical location, comorbidity, patient preference, and multidisciplinary judgment. However, these variables are often interpreted subjectively and may not fully capture tumor biology. AI could help integrate EUS morphology, radiomics, cross-sectional imaging, Ki-67 assessment, lesion growth kinetics, biochemical function, and patient-level risk factors into a more individualized prediction model. Such tools may eventually help distinguish patients suitable for surveillance, those requiring surgery, and those who may benefit from EUS-guided ablation as an organ-preserving alternative [22,28-31].

For functioning tumors, particularly insulinomas, AI-assisted selection could focus on confirming benign imaging behavior, defining lesion accessibility, estimating ablation feasibility, and predicting the likelihood of durable symptom control. For small non-functioning pNETs, the role may be even more important because the decision between surveillance, surgery, and ablation is often uncertain. In this group, AI could potentially identify low-risk tumors appropriate for local therapy while flagging lesions with imaging or clinical features suggestive of aggressive biology, higher grade, or metastatic potential. This would be particularly useful for tumors in the 1-2 cm range, where management remains individualized and where treatment decisions may be influenced by patient anxiety, surgical risk, and local expertise [2,3,13]. In this context, AI may support several stages of the EUS-guided ablation pathway, from detection and characterization to patient selection, procedural planning, ablation assessment, and follow-up (Table 1).

**Table 1:** Potential roles of artificial intelligence (AI) across the endoscopic ultrasound (EUS)-guided ablation pathway for pancreatic neuroendocrine tumors (pNETs)

Stage	Potential AI role
Detection	Automated identification of small pancreatic lesions on EUS
Characterization	Differentiation of pNETs from other solid pancreatic lesions
Grading prediction	Prediction of tumor grade or Ki-67 category using imaging features
Risk stratification	Estimation of malignant potential, growth risk, or progression risk
Patient selection	Supporting individualized choice between surveillance, surgery, or EUS-guided ablation
Procedural planning	Assessment of lesion size, location, duct proximity, and vascular relationships
Ablation assessment	Estimation of ablation completeness and residual viable tumor
Follow-up	Detection of residual disease, recurrence, or interval progression

AI may also support procedural planning by improving pre-ablation mapping. In principle, automated or semi-automated systems could measure lesion diameter and volume, define the relationship to the main pancreatic duct, estimate distance from major vessels, and support safe needle trajectory planning. Integration with contrast-enhanced EUS, elastography, CT, or MRI may further refine assessment of tumor vascularity and local anatomy. Although these applications remain largely investigational, they are relevant because incomplete ablation, duct injury, pancreatitis, and recurrence may be influenced by lesion size, location, vascularity, and proximity to critical structures [14-18,24].

**Figure 1:**
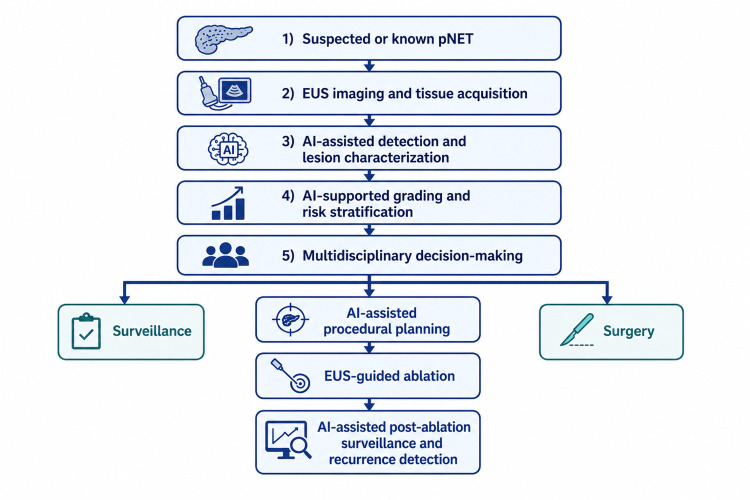
Proposed artificial intelligence (AI)-assisted endoscopic ultrasound (EUS)-pathway for pancreatic neuroendocrine tumors (pNETs) Figure created by the authors using Microsoft PowerPoint (Microsoft Corporation, Redmond, WA, USA).

Post-ablation surveillance represents another logical area for AI development. Current follow-up after EUS-guided ablation is not standardized and may rely on EUS, CT, MRI, biochemical response in functioning tumors, and clinical monitoring. AI-assisted imaging analysis could help detect residual enhancing tissue, quantify lesion involution, identify early recurrence, and standardize response assessment across centers. In insulinomas, AI models could theoretically combine imaging response with biochemical and symptomatic data to estimate the probability of durable remission. In non-functioning pNETs, AI-supported surveillance may be useful for detecting subtle progression after incomplete ablation or for identifying patients who should be referred for surgery [15,18,24]. A proposed AI-assisted EUS pathway integrating diagnosis, risk stratification, treatment selection, procedural planning, and post-ablation surveillance is illustrated in Figure 1.

At present, these applications remain conceptual and extrapolated from diagnostic AI and radiomics studies. Dedicated prospective studies are required before AI can be used to guide ablation eligibility or surveillance intensity in routine practice.

Ultimately, the value of AI will depend on whether it can improve clinically meaningful outcomes rather than simply increase diagnostic accuracy. For AI-assisted EUS-guided ablation to become clinically relevant, future studies must demonstrate better patient selection, fewer unnecessary operations, lower recurrence after ablation, earlier detection of residual disease, and safer procedural planning. Therefore, AI should be viewed as an enabling technology within a precision-endoscopy framework, rather than as a standalone solution [19,20,24].

Limitations and future directions

Despite growing interest in EUS-guided ablation for pNETs, the evidence base remains limited. Most studies are retrospective or observational, include relatively small cohorts, and vary in patient selection, tumor characteristics, ablation devices, energy settings, and follow-up protocols. Definitions of technical success, clinical response, radiological response, recurrence, and treatment failure are also inconsistent. These limitations make it difficult to determine the true durability of EUS-guided ablation, particularly for non-functioning pNETs, where long-term oncological control is harder to assess than symptom resolution in insulinomas [14-18].

The evidence supporting AI in this setting is even less mature. Although AI-assisted EUS and radiomics models have shown encouraging diagnostic and grading performance, most studies remain retrospective, single-center, and based on curated imaging datasets. External validation, prospective testing, real-time implementation, and assessment across different EUS platforms, operators, and institutions remain limited. Few studies have evaluated whether AI improves clinically meaningful outcomes, such as treatment selection, avoidance of unnecessary surgery, recurrence reduction, or earlier detection of residual disease [19-24,28-31].

Recent literature further highlights both the progress and the remaining evidence gaps in this field. Updated ENETS guidance provides contemporary management recommendations for non-functioning pNETs and reinforces the need for individualized decision-making in small lesions [32]. Recent prospective multicenter data also support the safety and effectiveness of EUS-radiofrequency ablation in selected functioning and non-functioning pNETs [33]. In parallel, emerging CT radiomics and machine-learning studies suggest potential value for non-invasive pNET grading [34], while externally validated multimodal AI models for solid pancreatic lesions provide stronger proof-of-concept for AI-assisted EUS diagnosis, although these findings should not be directly extrapolated to pNET-specific ablation selection without dedicated validation [22].

Future research should therefore focus on multicenter prospective studies that combine standardized EUS image acquisition, EUS-guided tissue sampling, cross-sectional imaging, pathology, procedural data, and long-term follow-up. Dedicated pNET registries would be particularly valuable for developing and validating AI models that predict tumor grade, progression risk, ablation feasibility, recurrence, and need for surgery. Explainable AI systems will also be important to ensure that clinicians can understand, verify, and safely apply model outputs in real-world decision-making [19,24,28].

Recent evidence further supports the need for continued refinement of both EUS-guided ablation and AI-based risk assessment. A recent prospective international multicenter study reported encouraging safety and effectiveness of EUS-RFA for both functioning and non-functioning pNETs, supporting its role as an organ-preserving option in selected patients [33]. In parallel, recent radiomics and machine-learning studies continue to suggest that imaging-based AI models may help predict tumor grade and biological risk, although these approaches still require stronger external validation before routine clinical adoption [34].

From a therapeutic perspective, future trials should compare EUS-guided ablation with active surveillance and surgery in carefully defined patient groups, particularly small insulinomas and low-risk non-functioning pNETs. Standardized reporting of tumor characteristics, ablation technique, adverse events, radiological response, biochemical outcomes, quality of life, recurrence, and long-term survival will be essential. Ultimately, the goal should not be to replace clinical expertise, but to integrate AI into a precision-endoscopy pathway that improves consistency, safety, personalization, and long-term outcomes for patients with pNETs [15,18,24].

## Conclusions

EUS-guided ablation represents a promising minimally invasive option for selected patients with pNETs, particularly small functioning insulinomas and carefully selected low-risk non-functioning lesions. Its main appeal lies in the potential to provide local tumor control while avoiding the morbidity of pancreatic surgery in patients for whom resection may be excessive, high risk, or undesirable. AI may further refine this evolving field by enhancing EUS-based lesion characterization, grade prediction, risk stratification, patient selection, procedural planning, and post-ablation surveillance. However, both EUS-guided ablation and AI-assisted decision support remain dependent on careful validation, multidisciplinary interpretation, and long-term outcome data. At present, AI should be viewed as an adjunct to expert judgment rather than a replacement for clinical, pathological, and radiological

Future integration of AI into EUS-guided ablation pathways may support a more personalized model of care in which patients are selected not only by tumor size and symptoms, but also by predicted biology, technical feasibility, treatment risk, and likelihood of durable response. This precision-endoscopy approach has the potential to improve individualized management of pNETs while maintaining patient safety and oncological caution.
